# Encapsulation and Protection of Omega-3-Rich Fish Oils Using Food-Grade Delivery Systems

**DOI:** 10.3390/foods10071566

**Published:** 2021-07-06

**Authors:** Vishnu Kalladathvalappil Venugopalan, Lekshmi Ramadevi Gopakumar, Ajeeshkumar Kizhakkeppurath Kumaran, Niladri Sekhar Chatterjee, Vishnuja Soman, Shaheer Peeralil, Suseela Mathew, David Julian McClements, Ravishankar Chandragiri Nagarajarao

**Affiliations:** 1 Centre for Marine Living Resources and Ecology (CMLRE), Ministry of Earth Sciences, Kochi 682508, India; vishnukalladath@gmail.com; 2 Central Institute of Fisheries Technology (CIFT), Indian Council for Agricultural Research, Matsyapuri P.O, Kochi 682029, India; lekshmirgcof@gmail.com (L.R.G.); ajeeshaksa@gmail.com (A.K.K.); niladri_icar@hotmail.com (N.S.C.); shaheerbb@gmail.com (S.P.); suseela1962@gmail.com (S.M.); cnrs2000@gmail.com (R.C.N.); 3 Department of Chemical Oceanography, School of Marine Sciences, Cochin University of Science and Technology, Cochin 682016, India; vishnujasiva@gmail.com; 4 Department of Food Science, University of Massachusetts, Amherst, MA 01003, USA

**Keywords:** marine lipids, polyunsaturated fatty acids, nanoencapsulation, microencapsulation, spray drying

## Abstract

Regular consumption of adequate quantities of lipids rich in omega-3 fatty acids is claimed to provide a broad spectrum of health benefits, such as inhibiting inflammation, cardiovascular diseases, diabetes, arthritis, and ulcerative colitis. Lipids isolated from many marine sources are a rich source of long-chain polyunsaturated fatty acids (PUFAs) in the omega-3 form which are claimed to have particularly high biological activities. Functional food products designed to enhance human health and wellbeing are increasingly being fortified with these omega-3 PUFAs because of their potential nutritional and health benefits. However, food fortification with PUFAs is challenging because of their low water-solubility, their tendency to rapidly oxidize, and their variable bioavailability. These challenges can be addressed using advanced encapsulation technologies, which typically involve incorporating the omega-3 oils into well-designed colloidal particles fabricated from food-grade ingredients, such as liposomes, emulsion droplets, nanostructured lipid carriers, or microgels. These omega-3-enriched colloidal dispersions can be used in a fluid form or they can be converted into a powdered form using spray-drying, which facilitates their handling and storage, as well as prolonging their shelf life. In this review, we provide an overview of marine-based omega-3 fatty acid sources, discuss their health benefits, highlight the challenges involved with their utilization in functional foods, and present the different encapsulation technologies that can be used to improve their performance.

## 1. Introduction

Regular intake of sufficiently high quantities of polyunsaturated fatty acids (PUFAs) has been needed to reduce the incidences of innumerable types of ailments and chronic diseases, including psoriasis, bowel diseases, mental illnesses, cancer, rheumatoid arthritis, cardiovascular diseases, diabetes, pulmonary disorders, coordination disorders, movement illnesses, obesity, and weak bones [1,2,3,4]. As a result, the consumption of foods rich in PUFAs is strongly encouraged by health agencies around the globe. Fish oil is one of the major dietary sources of PUFAs, especially eicosapentaenoic acid (EPA) and docosahexaenoic acid (DHA), which are believed to have particularly potent biological activities and health effects [5]. The overall PUFA profile of fish oils depends on fish species, sex, maturity, diet, and environment, which may impact their potential health benefits.

Incorporation of health-promoting omega-3 PUFAs into supplements, pharmaceuticals, and functional foods is limited. These unsaturated fatty acids are susceptible to oxidative degradation, therefore there is a chance of generation of undesirable aromas (“rancidity”), reducing consumer acceptance [6]. Moreover, some of the reaction products of lipid oxidation exhibit toxicity, which may lead to chronic health problems if they are regularly consumed over long periods [7]. As an example of the potential seriousness of this problem, scientists reviewing the nutritional value of fish oil supplements in New Zealand reported that most of them were highly oxidized [8]. This study indicated the importance of investigating the oxidation state of commercial fish oil supplements to ensure they are safe to consume. Another challenge for the development of omega-3-enriched food and beverage products is the very low solubility of fish oils in water. Finally, the bioavailability of omega-3 PUFAs may be relatively low and variable depending on the form they are delivered in. For instance, bulk forms have been shown to be adsorbed more slowly and to a lower extent than emulsified forms [9].

These challenges can often be overcome using advanced encapsulation technologies, which involve converting the omega-3 PUFAs into colloidal forms, like liposomes, lipid droplets, or biopolymer particles, which are then incorporated into foods or beverages [10]. In some applications, these colloid materials can be converted into a powdered form to facilitate their handling, storage, and utilization, as well as to increase their resistance to oxidation [11]. This can be achieved using a variety of processing operations including spray, freeze, and fluidized bed drying [12]. The selection of an appropriate encapsulation technology can lead to significant improvements in the chemical stability, water-dispersibility, and bioavailability of omega-3 oils.

In this article, we begin by reviewing the possible health benefits and biological mechanisms of action of omega-3 PUFAs. We then highlight methods of extracting omega-3 oils from marine sources, and the main challenges connected with incorporating them into foodstuffs. The different kinds of encapsulation technologies available to create fluid and powdered forms of omega-3 oils are then reviewed. Finally, methods of characterizing the properties of encapsulated omega-3 oils are highlighted.

## 2. Marine Lipids—Physiological Significance and Potential Health Benefits

**Figure 1:**
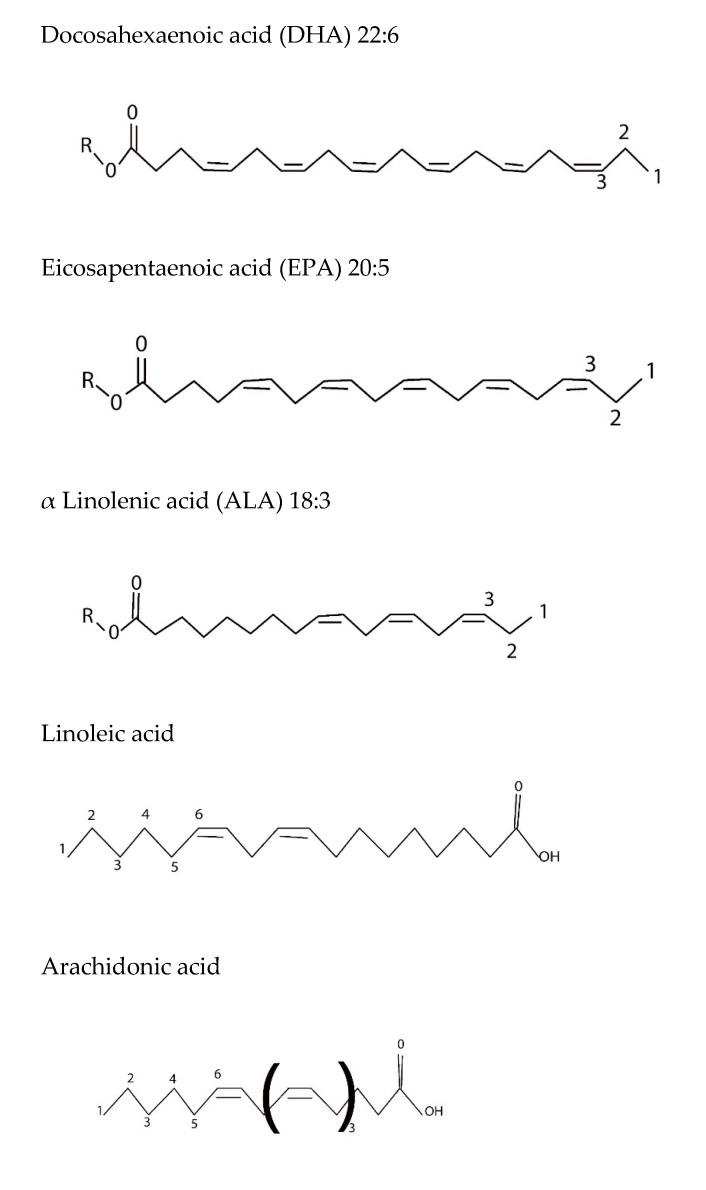
Structures of n-3 and n-6 fatty acids.

The potential health benefits of marine oils, especially fish oils rich in eicosapentaenoic acid (EPA) and docosahexaenoic acid (DHA), have been widely studied. Omega-3 fatty acids are long-chain PUFAs containing methylene-separated double bonds starting from the third carbon atom counted from the methyl-terminus (Figure 1). Physiologically, these fatty acids are reported to play an important role in a number of important biochemical processes. Research has also shown that adequate intake of these omega-3 fatty acids may help prevent the onset of a variety of chronic diseases. However, these fatty acids cannot be synthesized by humans and hence need to be obtained from the diet. A number of potential health benefits of omega-3 PUFAs from marine sources are highlighted in this section.

### 2.1. Inflammation

Inflammation, which is the body’s response to infection and cellular injuries, is mainly manifested by the production of inflammatory mediators like cytokines and reactive oxygen species (ROS), as well as the expression of adhesion molecules and arachidonic-acid-derived eicosanoids [13]. Intake of PUFAs competitively inhibits the metabolism of arachidonic acid [14], thereby increasing the production of omega-3-derived eicosanoids with anti-inflammatory effects. Studies have reported that EPA and DHA consumption also has anti-inflammatory effects by decreasing the expression of genes involved in inflammatory- and atherogenic-related pathways [15,16]. A recent meta-analysis of 31 randomized clinical trials reported that omega-3 PUFA supplementation reduced serum inflammation markers [17].

### 2.2. Oxidation

Reactive oxygen species such as hydroxyl radicals (^•^OH), superoxide anions (O_2_^−•^), and hydrogen peroxide (H_2_O_2_) are produced as an effect of aerobic respiration and substrate oxidation processes. Low levels of ROS are important for the normal functioning of cells, as they aid in critical biochemical processes such as intracellular messaging [18], immune responses [19], and defending against microorganisms [20]. However, high levels of ROS promote oxidative stress and induce metabolic malfunction and macromolecular damage [21]. Natural antioxidants can protect cells from the adverse effects caused by high levels of ROS [22]. Studies have shown that administration of natural antioxidants to mice reduced the activity of metabolic and antioxidant enzymes [23].

### 2.3. Lipid Profile

Clinical trials have shown that intake of omega-3 PUFAs significantly changes serum lipid profiles. For instance, consumption of omega-3 PUFAs was reported to decrease circulating plasma triacylglycerol (TG) levels [24]. When administered at pharmaceutical doses (3.4 g/day) for one month, there was around a 25–50% reduction in plasma triacylglycerols, as well as a decrease in hepatic very low density lipoprotein (VLDL) production and increase in VLDL clearance. A meta-analysis of 45 RCTs involving 2674 people with Type 2 diabetes reported that omega-3 PUFA supplementation had a favorable hypolipidemic effect being associated with significant reductions in blood low density lipoprotein (LDL), Very low density lipoprotein (VLDL), and triglyceride levels [25].

### 2.4. Cardiovascular Diseases

**Figure 2:**
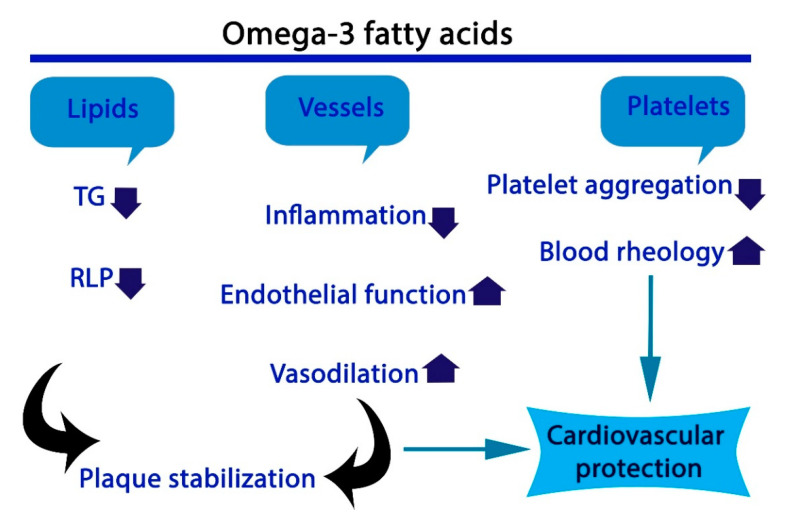
Role of omega-3 fatty acids in cardioprotection. (TG (triglycerides), RLP (Rmnant lipoprotein).

Several studies have reported an association between fish oil consumption and a reduced risk of cardiovascular diseases. For instance, a high intake of fish was reported to cause a reduction in risk factors associated with cardiovascular disease such as obesity, hypertension, and glycohemoglobin [26]. Similarly, an improvement in patients who had suffered a recent myocardial infarction was reported after their diet was supplemented with fish oil [27]. Based on the evidence of the potential cardioprotective effects of fish oil, the American Heart Association has recommended adults should eat fish at least twice per week [28]. A summary of the impacts of omega-3 PUFAs on cardiovascular diseases is shown in Figure 2. A meta-analysis of 13 randomized controlled trials found that omega-3 supplementation lowered the risk of myocardial infarction, coronary heart disease (CHD) death, and total CHD [29].

### 2.5. Thrombosis

The antithrombotic effects of fish oil have been known for many years [30] and demonstrated in a number of clinical trials [31]. Indeed, a meta-analysis of 15 randomized controlled trials in humans reported that omega-3 polyunsaturated fatty acids (PUFA) inhibit platelet aggregation [32].

### 2.6. Diabetes

Type 2 diabetes mellitus (T2DM) is a chronic lifestyle disorder where blood glucose levels are too high because of insulin deficiency or malfunction, which effects hundreds of millions of people worldwide [33]. Consumption of adequate quantities of omega-3 PUFAs has been reported to exhibit hypoglycemic and antidiabetic effects [34,35]. A meta-analysis of 12 randomized controlled trials reported that fish oil supplementation led to a more favorable blood lipids profile for patients with T2DM, but did not directly improve glucose control [36]. Despite there being no direct antihyperglycemic effects, fish oil supplementation did enhance lipid metabolism and suppress inflammation, thereby ameliorating insulin resistance.

### 2.7. Rheumatoid Arthritis

Fish oil supplementation has also been reported to have beneficial effects on rheumatoid arthritis by reducing various biomarkers for this disease [37]. Indeed, daily dietary consumption of fish oil allowed some patients to discontinue the use of non-steroidal anti-inflammatory drugs (NSAIDs) to treat their arthritis. Other studies have shown that consumption of a combination of fish oil and paracetamol have an anti-inflammatory effect in patients with rheumatoid arthritis [38]. A meta-analysis of 20 randomized controlled trials found that intake of ω-3 polyunsaturated-fatty-acid-rich fish oils played a major role in reducing the effects of rheumatoid arthritis [39].

### 2.8. Ulcerative Colitis

Ulcerative colitis is a chronic disease characterized by the influx and accumulation of neutrophils in the colonic mucosa [40]. Diets rich in omega-3 PUFAs have been shown to improve biomarkers associated with this disease [41,42]. A meta-analysis of 12 randomized controlled trials found that there was a significant relationship between dietary long-chain n-3 PUFAs and the risk of ulcerative colitis [43].

## 3. Recommended Intake of DHA and EPA

The potential health benefits associated with consumption of omega-3 fatty acids have led to a substantial increase in the number of fish oil supplements and fortified food products available on the market [44]. Based on the evidence from clinical trials and epidemiology studies, many governments and scientific organizations have set dietary guidelines that specify the recommended daily intake of EPA and DHA. For instance, the Institute of Medicine at the National Institute of Health (NIH) in the USA recommends an adequate intake of 1.1 to 1.6 g per day of omega-3 oils for adults. The European Academy of Nutritional Sciences (EANS) recommends an average intake of 0.2 g of omega-3 oil per day [45]. The International Society for the Study of Fatty Acids and Lipids (ISSFAL) and the American Heart Association recommends consumption of adequate fatty fishes through daily diet [28].

According to Rimm et al. [28] there should be a suitable balance between the omega-6 to omega-3 fatty acids in the food. They recommended that the ratio of omega-6 to omega-3 PUFAs should not exceed 4:1 for enhancing their bioavailability and metabolism. However, this ratio has risen to about 10:1 because of the elevated consumption of vegetable fats and oils rich in omega-6 PUFAs [46]. The joint FAO/WHO (Food and Agriculture Organization/World health Organization) Expert Consultation on Fats and Oils in Human Nutrition suggested that individuals with linoleic to linolenic acid ratios in excess of 10:1 should consume more foods rich in omega-3 PUFAs, mainly sea foods [14].

## 4. Extraction and Characterization of Fish Oil Lipids

### 4.1. Extraction Methods

#### 4.1.1. Traditional Methods

Extraction of fish oils can be achieved from whole fish, specific lipid-rich organs, or fish waste. Traditionally, fish oil extraction is carried out using conventional solvent-based methods with a combination of chloroform and methanol used as organic solvent [47]. Both polar and non-polar lipids can be isolated using this method. Initially, the fish tissue is ground with a combination of chloroform and methanol (2:1 ratio). Water can be added for making two separate layers. The bottom chloroform layer contains both polar and nonpolar lipids and the top methanolic and water layer contains all the other materials. After collection of the chloroform layer, solvent can be evaporated leaving the lipids. This method has been applied to both fish muscle and liver to extract omega-3 PUFAs [47]. For some fatty fish species, the majority of oil can be removed using physical methods, such as hydraulic pressing or heat extraction, but then the remainder of the oil may still be removed using organic solvent extraction.

#### 4.1.2. Green Methods

The isolation of fish oils using organic solvents has a number of drawbacks: (i) high temperatures degrade heat-sensitive omega-3 PUFAs, (ii) toxic organic solvent residues may remain within the final product, and (iii) organic solvents cause environmental pollution [48]. For this reason, more gentle, safer, and environmentally friendly green chemistry approaches are being developed to extract them, such as supercritical fluid-, enzyme-, microwave-, and ultrasound-assisted extraction methods [48,49]. At present, however, supercritical fluid extraction (SFE) is the most widely used of these green methods for the extraction of omega-3 marine oils because it can economically produce oils of high purity and yield without the need for high temperatures or organic solvents.

### 4.2. Characterization Methods

The nutrition, safety, and physicochemical properties of fish oils depend on their chemical composition and so it is important to have appropriate analytical methods to measure their composition. Gas chromatography (GC), often in combination with mass spectrometry (MS), is widely used for the analysis of non-polar lipids in fish oil samples [50]. Initially, the oils are chemically treated so that the triacylglycerols are converted into free fatty acid methyl esters (FAMEs) that are volatile and can be separated using appropriate GC columns. This leads to a chromatogram containing a series of peaks that correspond to fatty acids of different chain lengths and degrees of unsaturation. Traditionally, the fatty acids are quantified by measuring the areas under the peaks and identified by comparison with known standards. More detailed analysis can be performed by coupling the gas chromatography instrument with a mass spectrometer (GC-MS) [50]. In addition, important information about the types of lipids present in fish oils can be obtained using advanced high-resolution nuclear magnetic resonance (NMR) methods [51]. Information about the type of lipids present, such as triacylglycerols, diacylglycerols, monoacylglycerols, free fatty acids, and phospholipids can be obtained using thin layer chromatography (TLC) analysis [52]. These analytical methods are widely used in the emerging field of lipidomics, whose aim is to elucidate the structure and function of the many different kinds of lipid molecules present in biological samples [53].

## 5. Challenges to Fish Oil Incorporation into Foods

As mentioned earlier, there are numerous challenges that have to be overcome before fish oils can successfully be introduced into commercially functional foods and supplements. Fish oil consists of long-chain triacylglycerols that have an extremely low solubility in water because of the hydrophobic effect. Consequently, they cannot simply be mixed with water and usually have to be converted into a colloidal form before they can be incorporated into aqueous-based food and beverage products.

Fish oil is highly prone to rapid lipid oxidation because of the multiple double bonds (-C=C-) in omega-3 PUFAs [54]. The rate and extent of lipid oxidation is accelerated by certain environmental factors, such as light, heat, oxygen, transition metals, and some enzymes [55]. The oxidation of fish oils produces reaction products that have undesirable aromas (rancidity) that makes them unacceptable to consumers [56]. The rate at which fish oils oxidize during storage therefore has a major impact on their shelf-life. In addition, some of the reaction products of lipid oxidation have been shown to be toxic [57], which may lead to undesirable health outcomes if oxidized fish oils are consumed regularly. Consequently, effective strategies need to be employed to inhibit the oxidation of fish oils in food and supplement products [58]. A wide range of approaches have been developed to achieve this goal including controlling storage conditions, utilizing packaging, or adding antioxidants and chelating agents [59]. In addition, advanced encapsulation technologies can also be utilized as described in Section 6.

Another potential challenge for some fish oil formulations is their highly variable oral bioavailability, which depends on the molecular form of the omega-3 fatty acids and the nature of the surrounding food matrix [60]. The main cause of this effect is the relatively low solubility of omega-3 oils in the aqueous gastrointestinal fluids inside the human gut, as well as their susceptibility to chemical degradation during passage through the gut. After ingestion, fish oils pass through the mouth, esophagus, stomach, and small intestine where they may be absorbed. They are therefore exposed to a variety of digestive enzymes, pH conditions, mechanical forces, flow profiles, mineral ions, bile salts, and other gastrointestinal constituents. Bulk fish oils are converted into coarse oil-in-water emulsions in the mouth and stomach, whereas emulsified oils are already in a colloidal state prior to ingestion. Gastric and pancreatic lipases then adsorb to the surfaces of the lipid droplets and initiate lipid digestion, which involves converting triacylglycerols (TAGs) into free fatty acids (FFAs) and monoacylglycerols (MAGs). The resulting digestion products (FFAs and MAGs) then interact with endogenous bile salts and phospholipids to form mixed micelles that carry the lipids through the mucus layer to the surfaces of the epithelium cells where they can be absorbed. Studies have shown that the bioavailability of omega-3 oils is higher when they are ingested in an emulsified form than in a bulk form [61], which can be attributed to the greater surface area available for lipases to adsorb in emulsions. Consequently, controlling the initial colloidal state of fish oils, such as the composition, size, and surface characteristics of the particles they are encapsulated within, can have a major impact on their bioavailability profiles. In addition, the bioavailability of omega-3 oils can be enhanced by incorporating absorption enhancers into supplement formations, which increase the permeability of the epithelium cells [62].

The challenges associated with the low water-solubility, poor chemical stability, and low/variable bioavailability of omega-3 PUFAs can often be overcome using advanced encapsulation technologies.

## 6. Encapsulation

In this section, we examine some of the approaches that have been developed to encapsulate fish oils into colloidal particles that can improve the dispersibility, stability, and bioavailability of omega-3 PUFAs. The major techniques were compiled in Table 1. 

### 6.1. Encapsulation Technologies

#### 6.1.1. Liposomes

**Figure 3:**
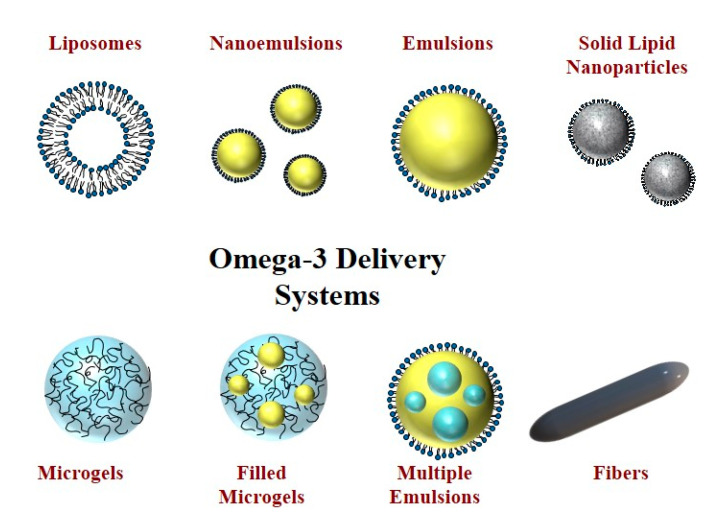
Examples of colloidal systems that can be used to encapsulate, protect, and deliver omega-3 PUFAs.

Liposomes are typically comprised of phospholipids organized into bilayer structures [63]. Commonly, they contain one or more bilayers that are organized into concentric rings (Figure 3). They therefore contain both non-polar and polar regions and so can be used to encapsulate hydrophilic and hydrophobic substances. A variety of different preparation methods are available to assemble liposomes, including solvent evaporation, injection, and microfluidization methods, which vary in their commercial potential [64]. Fish oil can be incorporated between the non-polar tails of the phospholipids. Alternatively, omega-3-rich phospholipids can be used to assemble the liposomes. Some studies have shown that encapsulation of oils within liposomes improves their oxidative stability during storage [73]. One of the disadvantages of this technology is that the phospholipids are relatively expensive, liposomes are often difficult to produce on a large scale, and they have low physical stability in complex food matrices.

Some studies highlighted that adding nano-encapsulated fish oil into some food products gave better stability and sensory characteristics than food fortified with free fish oil [74]. Rasti et al. [75] investigated liposome formulations with omega-3-rich fish oils and their oxidative stability. This study showed that PUFAs encapsulated in nanoliposomes (d = 50–200 nm) were more oxidatively stable than those encapsulated in liposomes (d > 200 nm), which was attributed to differences in the composition, size, and charge of the colloidal particles [75]. In particular, it was reported that producing liposomes without using organic solvents helped to protect them from oxidation. In the recent studies shrimp oil was also encapsulated using nanoliposomes using innovative methods such as microfluidization and ultrasonication. Developed nanoliposomes exhibited high encapsulation efficiency and oxidative stability [73].

#### 6.1.2. Emulsions and Nanoemulsions

Oil-in-water emulsions or nanoemulsions are widely acceptable methods for encapsulating fish oils [65]. These experiments consist of small emulsifier-coated oil droplets dispersed within water (Figure 3). The mean droplet diameter is below 100 nm for nanoemulsions but above this value for emulsions [66]. The smaller size of the droplets in nanoemulsions can lead to appreciable improvements in the bioavailability and physical stability of omega-3 oil formulations. Emulsions and nanoemulsions are usually formed by homogenizing an oil and water phase together in the presence of an emulsifier. A number of different homogenization devices are available including high shear mixers, colloid mills, high pressure valve homogenizers, microfluidizers, and sonicators. These devices vary in their operating principles, costs, throughput, and versatility. Recently, there has been interest in extending the functionality of conventional emulsions by using high oil contents (high-internal-phase emulsions) or by using particles as emulsifiers (Pickering emulsions) rather than surface-active molecules [76]. Many researchers have attempted various delivery systems for omega-3-rich fish oils. Walker et al. [77] reviewed studies of the efficacy of nanoemulsions-based delivery systems for adding omega-3 polyunsaturated lipids into food stuffs. Adding the PUFAs into small lipid droplets was found to enhance their water-dispersibility, physical stability, and bioavailability.

#### 6.1.3. Solid Lipid Nanoparticles and Nanostructured Lipid Carriers

Solid lipid nanoparticles (SLNs) and nanostructured lipid carriers (NLCs) are structurally similar to nanoemulsions but the lipid phase is either fully or partially crystallized (Figure 3) [67]. The crystallization of the lipid phase can improve the stability of encapsulated substances by slowing down the diffusion of pro-oxidants, thereby retarding their ability to interact with the omega-3 oils [68]. SLNs and NLCs can be formed using similar methods to those used for creating emulsions or nanoemulsions but homogenization is carried out above the melting point of the lipid phase. The preparation is then chilled below the crystallization temperature to stimulate a liquid-to-solid transition in the lipid phase. These systems must be judiciously formulated to avoid exclusion of the bioactives or accumulation during droplet crystallization [78].

#### 6.1.4. Multiple Emulsions

Multiple emulsions, which are also referred to as double emulsions, have a more complex structure than conventional emulsions (Figure 3). They mainly fall into two categories depending on the relative spatial location of the different phases—water-oil-water (W/O/W) and oil-water-oil (O/W/O) emulsions [69]. The W/O/W type is the most appropriate for the encapsulation of fish oils. This type of multiple layer emulsion consists of small water droplets (W_1_) dispersed inside larger oil droplets (O), which are themselves dispersed within a continuous water phase (W_2_). Multiple emulsions have a number of potentially useful applications in the food industry, such as calorie reduction, fat reduction, flavor masking, controlled release, and protecting sensitive ingredients [79]. Double emulsions are usually formed using a two-step procedure: (i) a W/O emulsion is formed by homogenizing a water phase with an oil phase containing an oil-soluble emulsifier and (ii) this W/O emulsion is further homogenized with a water phase containing a water-soluble emulsifier. Typically, the intensity of the second homogenization step should be less than that of the first to avoid breakup of the system. In this case, fish oils would form an oil phase that is separated by two aqueous phases. This may be advantageous if hydrophilic antioxidants can be incorporated into the internal water phase.

#### 6.1.5. Microgels

Edible microgels are normally made up of small particles from food-grade proteins and/or polysaccharides (Figure 3) [70]. These particles contain a network of physically or chemically crosslinked biopolymer molecules. Typically, omega-3 oils would be emulsified first and then the small oil droplets would be incorporated into the microgels. Microgels can be made using a variety of methods, including injection, phase separation, and molding methods [80]. For instance, in the injection method the oil droplets and a gelling biopolymer (like alginate) are injected into a gelling solution (like calcium), which leads to the formation of microgels with oil droplets embedded inside. In the coacervation approach, which is an example of a phase separation method, the oil droplets are mixed with a solution that contains two oppositely charged biopolymers (like a cationic protein and anionic polysaccharide), which again leads to microgels with oil droplets inside [81]. After preparation, microcapsules can be collected by either centrifugation or filtration and then used in a wet form or converted into a powder [82]. Researchers have encapsulated fish oil within coacervates assembled from various combinations of biopolymers, including hydroxypropyl methylcellulose–maltodextrin and whey protein–gum arabic [83,84].

#### 6.1.6. Nanofibers

Nanofibers consist of long thin fibrous materials that are typically assembled from food-grade biopolymers, like proteins or polysaccharides (Figure 3). These nanofibers can sometimes be used to encapsulate and control the release of hydrophobic substances. For instance, researchers have used an electro-spraying method to encapsulate DHA in zein nanofibers, which improves its oxidative stability [71].

#### 6.1.7. Inclusion Complexes

This approach involves trapping bioactive molecules into a cyclic oligosaccharide, such as cyclodextrin, to form a molecular inclusion complex. In the case of fish oil, the non-polar tails of the fatty acids are trapped within the hydrophobic cavity formed by the cyclodextrin. Studies have shown that encapsulation of fish oil in these complexes improves its oxidative stability [72]. Choi et al. [85] showed that fish oil could be encapsulated within cyclodextrin inclusion complexes at a high encapsulation efficiency.

## 7. Microencapsulation

In many applications, it is advantageous to convert a fluid fish oil formulation into a powdered form to improve its handling, storage, or stability, which is often referred to a microencapsulation [86,87]. Typically, the fish oil is emulsified and then mixed with an aqueous solution containing dissolved wall materials (Table 2 and Table 3). The resulting mixture is then dehydrated using an appropriate technology (Section 7.2). The powders resulting from these processes consist of microcapsules that contain numerous fish oil droplets embedded within a wall material [88].

### 7.1. Wall Materials

Wall material selection is very important. It plays a major role in the functional performance of the powder formed (flowability, packing, encapsulation efficiency, and chemical stability). Many factors influence the selection of an appropriate wall material for a particular application. The wall materials should be soluble in water and form a low viscosity fluid that can be pumped if required, e.g., in spray drying or electro-spraying. The wall materials should also lead to the formation of a powder that has the required flowability, stickiness, dispersibility, and solubility characteristics, as well as the ability to inhibit the diffusion of gasses and undesirable chemical reactions. Many of these physical attributes are determined by the glass transition temperature of the substances used to form the wall materials. The wall materials are hard and brittle below this temperature (glassy state), but soft and pliable above this temperature (rubbery state). Other additives may also be required to ensure the proper performance of the powdered materials produced by microencapsulation technologies, including emulsifiers, plasticizers, and defoaming agents [89]. Some of the most commonly used wall materials in the food industry are highlighted here and summarized in Table 2 and Table 3:

- Carbohydrates: maltodextrin, sucrose, corn syrup solids, modified starch, gum arabic, agar, alginates, carrageenan, pectin, and chitosan.
- Proteins: skimmed milk powder, gelatin, sodium caseinate, and whey protein.

**Table 3:** Examples of materials that have been used as wall materials for microencapsulation of fish oil. WPI, whey protein isolate; CS, chitosan; MD, maltodextrin; GA, gum arabic; SPI, soy protein isolate.

Wall Materials	Percentage of Wall Materials	Encapsulation Efficiency	Reference
WPI + CS+ MD for tuna oil	CS (0.5, 1, 1.5% *w*/*w*) MD (1% *w*/*w*), WPI (10% *w*/*w*)	80–86%	[90]
WPI for fish oil	WPI (1:2), SPI (3:1)	WPI—97% SPI—93%	[91]
CS + lecithin for tuna oil	CS (0.2% *w*/*w*) Lecithin (1% *w*/*w*)	87%	[92]
WPI + MD	90:10, 50:50, 10:90	45–65%	[93]
GA WPI GA + WPI for cardamom oil	100 g 100 g GA + WPI(1:1) GA + WPI ( 3:1)	92% 69.2% 83.3% 74.3%	[94]

### 7.2. Microencapsulation Technologies

Several technologies have been developed to convert fluid forms of fish oils into powdered forms, which differ in their commercial viability. At present, spray drying is typically the most commonly used because it is economical and can be carried out on an industrial scale. Nevertheless, some of the other approaches may have advantages for niche applications.

#### 7.2.1. Spray Drying

The spray drying of fish oil consists of several steps [95]: (i) formation of an oil-in-water emulsion by homogenization; (ii) mixing of emulsion with wall materials, which can be carried out before, during, or after homogenization; (iii) pumping the emulsion through a fine nozzle into a hot chamber leading to the formation of small droplets that are rapidly dried; and (iv) collection of the powder. In some cases, an additional agglomeration step may be included to improve the dispersibility of the resulting powders. The nature of the powders formed depends on the type of ingredients and processing conditions used and must be optimized depending on the application.

Spray drying has been used to encapsulate fish oils using numerous types of wall materials, including casein, lactose, dextrose, Maltodextrin, modified starch, glucose syrup, gum arabic, sugar beet pectin, and gelatin [96,97].

#### 7.2.2. Freeze Drying

Freeze drying can also be used to convert fluid omega-3 oil products into a powdered form [98]. This method involves two main processing steps: (i) the sample is first frozen to a temperature around −90 to −40 °C; (ii) the frozen sample is then dehydrated under vacuum leading to the production of a powder. In practice, freeze drying is not as widely used as spray drying in industry because it is more expensive, time consuming, and has a lower throughput [99]. Nevertheless, it does have some advantages over spray drying that may be beneficial for fish oil applications. In particular, there is no need for high temperatures, which reduces the susceptibility of the omega-3 oils to become oxidized during the microencapsulation process [100].

#### 7.2.3. Extrusion

Fluid forms of omega-3 oils can also be converted into a solid form using extrusion. This technology involves mixing molten wall materials with emulsified oil under high pressure, and then forcing them through a fine nozzle to produce solidified microcapsules [101]. This method is capable of large scale commercial production of microcapsules but it does have some disadvantages for fish oil microencapsulation. In particular, the high temperatures used may promote the oxidation of the fish oil during the manufacturing process and it involves high capital and energy costs [101]. There are a number of different extrusion approaches that can be used for microencapsulation purposes, including centrifugal extrusion, melt-injection, and melt-extrusion [102], which vary in their operating principles and the nature of the microcapsules produced.

#### 7.2.4. Electro-Spraying and Electro-Spinning

Fluid materials can be converted into powders using electro-spraying or electrospinning technologies [103]. In the food industry, these methods typically involve placing a solution of biopolymers into a syringe, then applying a strong electrical field, which pulls out the biopolymer solution. As the stream of solution moves from the syringe to a charged collector plate, the water is rapidly evaporated, leading to the formation of particles (electro-spraying) or fibers (electro-spinning) depending on the operating conditions used. Fish oil is first converted into an emulsified form, which is then mixed with the biopolymer solution prior to the electro-spraying or electro-spinning process [84].

## 8. Characterization of Encapsulated Microparticles

After microparticle preparation, it is essential to distinguish the properties of the microcapsules produced. The main factors that need to be characterized are the microstructure, encapsulation efficiency, percentage yield, particle size, loading capacity, bulk density, moisture content, tapped density, hygroscopicity, and oxidative stability. A variety of analytical techniques are employed to characterize the structure of the powders produced by microencapsulation [104,105]. Their microstructure can be evaluated using optical microscopy, scanning electron microscopy (SEM), or transmission electron microscopy (TEM). The physical state of the wall materials within the powders, such as whether they are crystalline or amorphous, can be established using X-ray powder diffraction or differential scanning calorimetry (DSC). DSC can also be used to measure the glass transition temperature (T_g_) of the wall materials, which is an important parameter determining their functional performance. Information about the chemical composition and molecular interactions in powders can be obtained using Fourier transform infrared (FTIR) or Raman spectroscopy.

The encapsulation efficiency (EE) is the ratio of oil trapped inside the wall material to the total initial concentration used. For practical applications, it is often important to ensure that the content of surface oil, which is the oil that is not trapped inside the wall material, should be as low as possible. This is because the surface oil is directly exposed to air so can easily undergo oxidation. Ideally, the surface oil content should be less than 0.1% (*w*/*w*) in a high quality powder produced by microencapsulation. The encapsulation efficiency can be calculated using the following relationship, Equation (1) is as follows:

EE = (m_TO_ − m_SO_/m_TO_) × 100 (1)

where m_TO_ is the total mass of oil and m_SO_ is the mass of the surface oil in the powder. The loading capacity (LC) provides a measure of the amount of oil encapsulated per unit mass of powder, Equation (2) is as follows:

LC = (m_E_/m_T_) × 100 (2)

where m_E_ is the mass of the encapsulated oil and m_T_ is the total mass of the powder. The percentage yield (PY) is a measure of the efficiency of powder production and collection during the microencapsulation process, Equation (3) is as follows:

PY = (m_T_/m_I_) × 100 (3)

where m_I_ is the initial mass of the materials (emulsified oil and wall materials) used to produce the powder.

The size of the microcapsules in the powder impacts the oxidative stability of the final product since it determines the surface area exposed to the surrounding air. Typically, the smaller the size, the larger the surface area, and the greater the exposure to air, which should lead to faster oxidation. However, the size of the microcapsules also impacts their other properties, such as their tendency to aggregate and their dispersibility in water. Consequently, it is important to establish the optimum size of the microcapsules for a particular application. The size of the microcapsules can be measured using static light scattering or microscopy methods, such as TEM (transmission electron microscopy), SEM (scanning electron microscopy) or confocal laser scanning microscopy (CSLM). CSLM is particularly useful for identifying the location of the oil in the powder since oil-soluble fluorescence dyes can be added.

The bulk density and tapped density are two vital parameters for the packaging, storage, and transport of powdered products and is calculated by determining the volume occupied by a known mass of powder before and after tapping. The bulk density of a powder sample is the ratio of the mass to the volume of an untapped powder sample. The tapped density is obtained by mechanically tapping a graduated cylinder containing the sample until little further volume change is observed [104]. Based on the results, the flowability and cohesiveness of the microparticle can be determined. Flowability and cohesiveness of powders can be termed by the Carr index (*CI*) and Hausner ratio (*HR*), respectively [106], Equations (4) and (5) are as follows:

*CI* (%) = *TD* − *BD*/*TD* × 100 (4)

*HR* (%) = *TD*/*BD* × 100 (5)

where, *TD* is the tapped density and *BD* is the bulk density.

The moisture content of the encapsulated powder plays an important role in determining the flowability, stickiness, cohesiveness, hygroscopicity, and storage life. This can be simply determined using oven drying methods, chemical methods (Karl Fisher titration), or spectroscopy methods (FTIR). The maximum moisture content of powders for food industry application should be around 3 to 4%. Hygroscopicity is the capacity of a powder to absorb water from the environment, which depends on its composition and structure. It also plays an important role in the reconstitution of the powder in water since it can lead to caking, which reduces dispersibility [107]. The hygroscopicity is typically measured by weighing a sample over time after it is placed in an environment with a known relative humidity, and can be expressed as grams of adsorbed moisture per 100 g of dry solids [108].

An important goal of encapsulation is to protect the bioactive material against oxidation by providing an oxygen barrier in the form of wall materials. The oxidative stability of fish oils encapsulated within powders is usually measured by storing them under controlled temperature and relative humidity conditions and periodically measuring markers of lipid oxidation, such as primary or secondary reaction products. For instance, conjugated dienes and peroxide value measurements can be used as indicators of primary reaction products, whereas thiobarbituric acid reactive substances (TBARS) and propanal can be used as indicators of secondary reaction products.

The dispersibility of the powders in water can be monitored by measuring how quickly the emulsified lipids are released after rehydration. This can be achieved by dispersing the powders in the measurement chamber of a light scattering instrument and measuring the change in the light scattering pattern over time [109]

## 9. Digestibility of Encapsulated Fish Oil

Encapsulation is regularly used to increase the stability, handling, and application of fish oils, but it is significant that it does not unfavorably affect their bioavailability. For instance, inserting emulsified fish oil within dietary fiber particles could reduce its digestion and absorption within the gastrointestinal tract. Conversely, a well-designed encapsulation system could actually increase the bioavailability of fish oils. Consequently, it is important to establish the impact of encapsulation on the digestibility of fish oils. This is usually achieved using standardized in vitro digestion models [110].

A number of studies have examined the impact of different encapsulation technologies on the digestibility of omega-3 oils. For instance, Klinkesorn and McClements [111] showed that coating fish oil droplets with chitosan did not inhibit their digestion and release under simulated gastrointestinal conditions. Chang et al. [112] showed that a chitosan coating can also impact the gastrointestinal fate of emulsified fish oils by protecting it from degradation by gastric enzymes, thereby leading to a more sustained release under intestinal conditions. Xu et al. [113] found that two anionic polysaccharides (pectin and xanthan gum) could increase the digestion of protein-coated fish oil droplets, which was attributed to their ability to inhibit droplet flocculation, thereby increasing the surface area for the lipase to adsorb. Chang and McClements [114] reported that a marine polysaccharide (fuicodan) impacted lipid digestion in fish oil-in-water emulsions stabilized by different emulsifiers (whey protein, casein, or Tween). This anionic polysaccharide also increased the rate of lipid digestion in the emulsions containing the protein-coated droplets for a similar reason. Gumus et al. [115] examined the impact of protein emulsifier type (lentil, pea, or fava bean protein) on the digestibility of fish oil-in-water emulsions using a simulated gastrointestinal tract and found that all of the lipid droplets were digestible. Finally, Qiu et al. [116] also examined the impact of protein emulsifier type (gliadin, caseinate, and whey protein) on the digestion of fish oil droplets and found that gliadin gave the slowest digestion rate. These results show that the digestibility of fish oil, and therefore its bioavailability, depend on designing colloidal delivery systems carefully.

## 10. Conclusions

Fish oil is an excellent dietary source of polyunsaturated fatty acids such as EPA and DHA, which have been shown to exhibit a diverse range of health benefits. For this reason, there has been growing interest in fortifying foods and beverages with these marine-derived omega-3 fatty acids. However, this is typically challenging because of the poor water solubility, chemical stability, and bioavailability characteristics of these oils. In this article, we have described a number of different encapsulation technologies that can be used to overcome these problems. We have also described the methods available to convert fluid forms of fish oil formulations into powders, which can be used to enhance the handling, storage, stability, and application. As mentioned earlier, a number of different colloidal delivery systems have been successfully used to encapsulate and protect omega-3-rich marine oils. However, from a practical point of view, emulsions and nanoemulsions are usually the most suitable for this purpose since they can easily be formulated using food-grade ingredients and existing processing operations, such as mixing and homogenization. Moreover, they can be formulated from a wide range of food-grade emulsifiers, such as proteins, polysaccharides, phospholipids, and surfactants, which provides considerable flexibility in designing their properties In future studies, it will be important to identify the optimum encapsulation technologies for specific food products. In addition, the economic viability and scalability of these processes must be elucidated before they can find widespread commercial adoption.

**Highlights**

- Fish oil is rich in health-promoting omega-3 polyunsaturated fatty acids (PUFAs)
- PUFAs are difficult to incorporate into foods due to low water-solubility and chemical stability
- Encapsulation technologies can be used to overcome dispersibility and stability issues
- Novel and conventional encapsulation technologies are reviewed.

## Figures and Tables

**Table 1:** Different kinds of delivery systems that have been used to encapsulate omega-3 fish oils.

Sl.No	Encapsulation Type	Details	Reference
1.	Liposomes	Commonly, they contain either one or more bilayers. They therefore contain both non-polar and polar regions and so can be used to encapsulate hydrophilic and hydrophobic substances.	[63,64]
2.	Solid lipid nanoparticles and nanostructured lipid carriers	Nanoemulsions are the widely acceptable methods for encapsulating the fish oils. These experiments consist of small emulsifier-coated oil droplets dispersed within water. The mean droplet diameter is below 100 nm for nanoemulsions but above this value for emulsions.	[65,66]
3.	Solid lipid nanoparticles and nanostructured lipid carriers	Solid lipid nanoparticles (SLNs) and nanostructured lipid carriers (NLCs) are structurally similar to nanoemulsions but the lipid phase is either fully or partially crystallized. The crystallization of the lipid phase can improve the stability of encapsulated substances by slowing down diffusion of pro-oxidants, thereby retarding their ability to interact with the omega-3 oils.	[67,68]
4.	Multiple emulsions	Multiple emulsions have a more complex structure than conventional emulsions. They mainly fall into two categories depending on the relative spatial location of the different phases—water-oil-water (W/O/W) and oil-water-oil (O/W/O) emulsions. The W/O/W type is the most appropriate for the encapsulation of fish oils.	[69]
5.	Microgels	Edible microgels are normally made up of small particles that are developed from food-grade proteins and/or polysaccharides. These particles contain a network of physically or chemically cross-linked biopolymer molecules. Typically, omega-3 oils would be emulsified first and then the small oil droplets would be incorporated into the microgels.	[70]
6.	Nanofibers	Nanofibers consist of long thin fibrous materials that are typically assembled from food-grade biopolymers, like proteins or polysaccharides. These anofibers can sometimes be used to encapsulate and control the release of hydrophobic substances.	[71]
7.	Inclusion complexes	This approach involves trapping bioactive molecules into a cyclic oligosaccharide, such as cyclodextrin, to form a molecular inclusion complex. In the case of fish oil, the non-polar tails of the fatty acids are trapped within the hydrophobic cavity formed by the cyclodextrin.	[72]

**Table 2:** Common materials used to encapsulate omega-3 PUFAs in food applications.

Encapsulant Materials		
Carbohydrates	Proteins	Lipids and Waxes
Native starches Modified starches Resistant starches Maltodextrins Gum acacia Alginates Pectins Carrageenan Chitosan	Sodium caseinate Whey proteins Isolated whey proteins Soy proteins Gelatins Zein Albumin	Vegetable fats and oils Hydrogenated fats Palm stearin Camauba wax Bees wax Shellac Polyethylene glycol

## Data Availability

The study did not report any data.
